# Induced Genetic Variations in Stomatal Density and Size of Rice Strongly Affects Water Use Efficiency and Responses to Drought Stresses

**DOI:** 10.3389/fpls.2022.801706

**Published:** 2022-05-25

**Authors:** Mutiara K. Pitaloka, Robert S. Caine, Christopher Hepworth, Emily L. Harrison, Jennifer Sloan, Cattleya Chutteang, Chutima Phunthong, Rangsan Nongngok, Theerayut Toojinda, Siriphat Ruengphayak, Siwaret Arikit, Julie E. Gray, Apichart Vanavichit

**Affiliations:** ^1^Faculty of Agriculture Kamphangsaen, Kasetsart University, Nakhon Pathom, Thailand; ^2^Plants, Photosynthesis and Soil, School of Biosciences, University of Sheffield, Sheffield, United Kingdom; ^3^Department of Agronomy, Faculty of Agriculture Kamphangsaen, Kasetsart University, Nakhon Pathom, Thailand; ^4^Rice Science Center, Kasetsart University, Nakhon Pathom, Thailand; ^5^National Center of Genetic Engineering and Biotechnology (BIOTEC), National Science and Technology Development Agency (NSTDA), Khlong Luang, Thailand

**Keywords:** mutant, stomata, rice, drought, WUE

## Abstract

Rice (*Oryza sativa* L.) is an important food crop relied upon by billions of people worldwide. However, with increasing pressure from climate change and rapid population growth, cultivation is very water-intensive. Therefore, it is critical to produce rice that is high-yielding and genetically more water-use efficient. Here, using the stabilized fast-neutron mutagenized population of Jao Hom Nin (JHN) - a popular purple rice cultivar - we microscopically examined hundreds of flag leaves to identify four stomatal model mutants with either high density (HD) or low density (LD) stomata, and small-sized (SS) or large-sized (LS) stomata. With similar genetic background and uniformity, the stomatal model mutants were used to understand the role of stomatal variants on physiological responses to abiotic stress. Our results show that SS and HD respond better to increasing CO2 concentration and HD has higher stomatal conductance (gs) compared to the other stomatal model mutants, although the effects on gas exchange or overall plant performance were small under greenhouse conditions. In addition, the results of our drought experiments suggest that LD and SS can better adapt to restricted water conditions, and LD showed higher water use efficiency (WUE) and biomass/plant than other stomatal model mutants under long-term restricted water treatment. Finally, our study suggests that reducing stomata density and size may play a promising role for further work on developing a climate-ready rice variety to adapt to drought and heat stress. We propose that low stomata density and small size have high potential as genetic donors for improving WUE in climate-ready rice.

## Background

Rice (*Oryza sativa* L.) is a major crop, providing food for billions of people globally (Redfern et al., 2012; Elert, 2014). However, its cultivation is water-intensive, requiring approximately 2,500: of water per kg of polished grain produced (Bouman, 2009). Most of the up taken water by a rice plant transpires through stomata pores. Therefore, rice’s low water-use efficiency is extremely vulnerable to climate change (Meyer et al., 2014; UNESCO, 2016; Yao et al., 2017; Caine et al., 2018). Drought and heat are two major threats to rice growth and development (Solomon et al., 2009; Jagadish et al., 2015; Korres et al., 2017; Kumar et al., 2017; Caine et al., 2018). Rice has one of the highest stomatal densities and one of the smallest stomatal size of all plant species (Bertolino et al., 2019). Leaf regulates gaseous exchange and transpiration with the environment *via* stomatal complexes, which predominantly develop in files on the epidermis in rice (Luo et al., 2012; Hepworth et al., 2018). Each stomatal complex consists of a pair of dumbbell-shaped guard cells surrounded by specialized epidermal cells called subsidiary cells, which may aid in increasing the efficiency of opening and closing the stomatal guard cells (Chen et al., 2017; Raissig et al., 2017). Because the important functions of stomata are gas exchange and transpiration, altering stomatal traits, such as density and size, are hypothesized to be the key to improving water use efficiency (WUE) (Bertolino et al., 2019).

Plants capture CO_2_ for photosynthesis by increasing stomatal aperture with the concurrent release of water, enabling plant cooling when temperatures are high (Zeiger et al., 1987; Mustilli et al., 2002; Caine et al., 2018). During short-term drought, reductions in stomatal aperture restrict CO_2_ uptake and water release, leading to increased plant temperature (Raven, 2014; Hepworth et al., 2015). A study of 49–62-day-old rice under the same water stress conditions found that low stomatal density rice was cooler than the higher stomatal density control rice (Caine et al., 2018).

Throughout the entire cycle of plant growth, stomatal development is adjusted to fit the surrounding environment, often by modifying the density and size of stomata on the plant epidermis (Mustilli et al., 2002; Casson and Gray, 2008; Caine et al., 2018). On the one hand, exposure to prolonged high light intensity, high humidity, or low atmospheric CO_2_ concentrations often leads to increased stomatal density (Casson et al., 2009; Tricker et al., 2012). On the other hand, drought, low light, or increased atmospheric CO_2_ concentration reduces stomatal density (Hetherington and Woodward, 2003; Casson and Gray, 2008; Hamanishi et al., 2012; Engineer et al., 2014). When the temperature is increased, the stomatal density is reduced in *Arabidopsis* (Crawford et al., 2012), but increased in rice (Caine et al., 2018).

Recent research on stomatal responsiveness to light across a range of plant species showed that the small dumbbell-shaped stomata of grass species respond faster than large dumbbell-shaped stomata. For plants with elliptical-shaped stomata, size does not correlate with stomatal response speed (McAusland et al., 2016; Lawson and Vialet-Chabrand, 2018). A recent study of the SLAC1-deficient rice mutant revealed that it exhibited significantly faster stomatal opening upon an increase in light irradiance, which led to faster photosynthetic induction (Yamori et al., 2020). By altering stomatal aperture in response to light more rapidly, the *g*_*s*_ of small dumbbell-shaped stomata was more rapidly altered, leading to improved WUE (iWUE). When the stomatal densities of grass species with small dumbbell-shaped stomata were assessed, no negative correlation was found between stomatal size and density, except for rice. Translational research related to the regulation of stomatal development in grasses is rapidly expanding, with a number of researchers analyzing the physiological effects of altering stomata (Hughes et al., 2017; Raissig et al., 2017; Caine et al., 2018; Schuler et al., 2018). In *Arabidopsis* and rice, stomatal density is controlled by a single gene that regulates the development of epidermal cells (Sugimoto et al., 2013; Bertolino et al., 2019). Overexpression of EPF1 markedly reduced stomatal density and improved iWUE in barley and rice (Hughes et al., 2017; Caine et al., 2018). Alternatively, the CRISPR technology generated a specific knockout on an ortholog EPFL9 to successfully develop a mutant with reduced stomatal density (Yin et al., 2017). Therefore, variation in stomatal density and size can alter assimilation (*A*) and *g*_*s*_, leading to changes in iWUE.

The success of conventional breeding and the marker-assisted selection relies heavily on available genetic variation. The depletion of genetic diversity is therefore a crucial limiting factor for marker-assisted breeding in helping to feed the increasing world population. It has been known that mutagenesis, induced by radiation or chemicals, can rapidly trigger structural and nucleotide changes in the genome, which consequently induce both random and spontaneous mutations that may lead to phenotypic gain and be used in breeding programs (Ruengphayak et al., 2015; Kamolsukyeunyong et al., 2019). In this study, we used a stabilized M6 generation of fast-neutron bombarded Jao Hom Nin (JHN) rice mutant core collection resulting from several reverse and forward selections for distinct genetic variation for resistance to biotic and abiotic stresses and grain qualities. Such a mutant core collection represented the most diverged germplasm derived from a single mutagenized population. This gave us a better chance to search for the relics of induced genetic variation for other morphological traits, such as stomatal density and size. Here, we microscopically screened 216 M_6_ mutant core collection (MCC) for rice mutants with unique stomatal density and size alterations. We selected four stomatal mutants representing extreme stomatal traits for high density (HD), low density (LD), large size (LS), and small size (SS). We also examined how such alterations in stomatal size and density impacts gas exchange, responses to drought, and WUE in an attempt to generate new rice variety traits that can combat climate for the coming years and decades.

## Materials and Methods

### Identification of Stomatal Model Mutants

#### Base Population

Jao Hom Nin is a photoperiod-insensitive, low amylose, and purple rice cultivar developed by Rice Science Center, Kasetsart University, Thailand. In order to regenerate useful genetic variability, a large batch of 100,000 purified viable seeds (M_0_) of the purple rice was bombarded with 33 Gy fast-neutron followed by four generations (M_1_–M_4_) of isolating viable mutants by self-pollination. As of the M_4_, forward and reverse screenings for useful genetic alteration were undertaken as previously described (Ruengphayak et al., 2015). In the M6 generation, 216 mutants with distinct phenotypes for resistance to biotic and abiotic stresses and grain qualities were collectively called M_6_ MCC. As of the M_6_ MCC, it was maintained by self-pollination and pure-line selection. The fast-neutron bombarded mutant population was maintained in a sufficiently irrigated, chemical-free paddy field at Kasetsart University, Kamphaeng Saen Campus in summer 2017 when the average daily temperature was approximately 36°C.

#### Microscopic Observation of Stomatal Traits

The 216 M_6_ MCC lines were field-grown in summer 2017 at Kasetsart University. Fully expanded flag leaves were collected from three plants within each line. Then, three leaf segments of each leaf were imprinted on glass slides for further observations under light microscopes. Stomatal size (guard cell length) and density were measured using light microscopy on three biological replicates per line. Stomatal density was counted within each view of the taken picture and converted to mm^2^ (detail described below). From phenotypic observation, four distinct stomatal trait variants showing either high density (JHN2447, HD) or low density (JHN8756, LD) stomata and small sized (JHN3117, SS) or large sized (JHN826, LS) stomata, collectively called stomatal model mutants, were selected for further physiological responses under abiotic stresses. Leaf sections from the adaxial and abaxial leaf surfaces were imprinted on glass slides by applying dental resin (Coltene Whaledent, Switzerland) applied in the region of maximum leaf width and left to set before removing the leaf and applying clear nail varnish to the resin. Stomatal counts were determined from nail varnish impressions and the pictures were captured from the middle area of leaves from at least eight plants per genotype. Cell count was conducted on six fields of view (FOV), which is a picture taken from 40× microscope magnification (2,048 × 1,536 pixels) per leaf. Epidermal imaging of the leaf was captured using camera-mounted light microscopes (Leica, DM750-ICC50 HD, Germany). Using ImageJ software (Fiji v. 1.51 μ, United States), stomata cells within each field of view were counted and converted into the total stomatal density per mm^2^. The stomatal complex was studied on the four stomatal model mutants: SS, LS, HD, and LD. From 10 randomly selected stomata from each FOV, the length from the tip to the base of guard cells were used to estimate the average stomatal size of each line. The total stomatal complex area was calculated by manually tracing around the outside of both the guard cells and subsidiary cells using the polygon tool of the ImageJ.

### A-Ci Measurement

This experiment aimed to understand the impact of stomatal size/density on carbon assimilation from CO_2_ enrichment from 100 up to 1,500 ppm. The experiment was conducted at the University of Sheffield, Sheffield, United Kingdom in May–July 2017. Mutant line seeds were brought from Thailand and germinated in Petri dishes for 7–8 days in a Sanyo growth cabinet, set to 12 h 26°C:12 h 24°C (light:dark) with 200 μmol m^–2^ s^1^ photosynthetically active radiation (PAR). Seedlings were then transferred to 13D pots and grown in Conviron growth cabinets (Controlled Environments Ltd., Winnipeg, MB, Canada) at 12 h 30°C: 12 h 24°C (light: dark), PAR 1,000 μmol m^–2^ s^–1^, and 60% relative humidity. The experiment was designed as a completely randomized design in five replications, comparing SS, LS, HD, LD, and JHN control. The infra-red gas analyzer (IRGA) was conducted on the first fully expanded flag leaf from the primary tiller using a LI-6400XT portable photosynthesis system. The chamber flow rate was set at 400 μmol s^–1^, leaf temperature at 32°C, and light intensity at 2,000 μmol m^–2^ s^–1^. *A* was measured for the following CO_2_ levels: 100, 200, 340, 480, 600, 800, 1,000, 1,200, and 1,500 ppm. The time allowed for *A* to stabilize before the measurements were automatically recorded was 10 min (Pandey et al., 2017).

### Leaf Gas Exchange Measurement at Steady State and Rapid Response to Dark/Light Transition Experiment

The experiment compared stomatal model mutants and JHN control to understand the stomatal closure and opening of the first flag leaves in response to rapid dark/light transitions. As previously described, all pot plants were grown until the first flag leaf emerged in a greenhouse condition between November 2017 and January 2018. The experiment was designed as a factorial in a completely randomized design with three replications, comparing two groups of stomatal variants. The first group compared stomatal size variants (SS, LS) and JHN wild type. LS and the second group compared stomatal density variants (HD and LD) and JHN wild type. IRGA measurements were conducted on the first fully expanded flag leaf from the primary tiller using LI-6400XT portable photosynthesis systems. The chamber flow rate was set at 400 μmol.s^–1^, leaf temperature at 32°C, reference [CO_2_] at 400 ppm, and light intensity at 2,000 μmol.m^–2^ s^–1^. Relative humidity inside the chamber was kept at 65–75% using a self-indicating desiccant. Steady-state measurements under these conditions were taken for *A* and stomatal conductance (*g*_*s*_), and iWUE was calculated from *A* divided by stomata conductance (*A*/*gs*). For light-dark-light response curves conducted in the greenhouse, plants were acclimatized at saturating light (2,000 μmol.m^–2^ s^–1^), followed by 10 min of complete darkness (0 μmol.m^–2^.s^–1^). Finally, saturating light was re-applied for the final 10 min. At each light intensity, 20 measurements for *g*_*s*_ were taken.

### Drought Experiments

Two drought experiments were undertaken to compare the four stomatal mutants and JHN control in short-term greenhouse and long-term outdoor conditions. The stomatal density of the stomatal model mutants and JHN control were immediately checked before the drought treatment (Figure 2 and Supplementary Table 2).

### Short-Term Drought Experiment

Seeds were germinated and grown in pots filled with 4.5 kg of uniformly mixed dried clay-loam paddy soil. Plants were grown in the High-Resolution Plant Phenotyping (HRPP) facility in November–December 2017 when the average temperature ranged between 30 and 40°C. Organic fertilizers were applied twice at the late seedling and the maximum tillering stages. Eight plants of each line were grown, and physiological and agronomic traits were determined on flag leaves between November and December 2017. These plants were grown on sufficient irrigation until 71 days after germination.

The HRPP housed a dark/light-adapted tunnel, Chlorophyll Fluorescent imaging system and automated irrigation system. Raw data were analyzed, and images were acquired using a plant data analyzer program (PSI).

a) **Drought condition:** Restricted irrigation began from complete water drainage right on 71 days after germination, when mutants and JHN reached the R_1–2_ reproductive stage (Counce et al., 2000). After drying for 5 days, 200 ml of water was irrigated every 2 days for up to 14 days. Then sufficient irrigation resumed up to harvest. Physiological and agronomic traits were collected on both sufficient and restricted irrigation schemes until harvest.
b) **Chlorophyll fluorescence:** For measurement taken under controlled condition, FluorCam was used to capture chlorophyll fluorescence images and to estimate the maximum quantum yield of PSII (*Fv/Fm*). Fluorescence chlorophyll imaging was set up without dark adaptation every morning (6 A.M.) for 5 days. Data analysis and image acquisition was conducted using Plantscreen data analyzer program version 3.1.0.18 (PSI, Brno, Czechia). On the first 5 days of soil drying, chlorophyll fluorescence was monitored daily using PSI Open FluorCam FC 800-O, applying PAM (Pulsed Amplitude Modulated) technology (FluorCam Operation Manual V2.1, PSI, Brno, Czechia). A high-intensity actinic light pulse was set at 1,800 μmol photon m^2^ s^–1^ to estimate the maximum quantum yield of PSII (F_v_/F_m_) of rice plants under sufficient and restricted irrigations. Soil water potential was monitored daily using a tensiometer.
c) **Agronomic traits:** Grain yield and biomass were collected at harvest and dried to 14% moisture content. Harvest index (HI) is the ratio of the grain yield and the total above-ground biomass yield. The percentage reduction on each agronomic trait is the ratio of the trait values in the restricted over the sufficient irrigations.

### Long-Term Drought Experiment

Rice seeds were pre-germinated on 200-well plastic plates. After 5 days, germinating seeds were sown 1–2 cm below soil surface into large concrete tanks located on the experimental field at Kasetsart University, Kamphang Saen Campus during February–May of summer 2019 when the average temperature was between 35 and 40°C. The round concrete tank is 1.2 m (diameter) × 0.8 m (height) and could hold 750 kg of paddy soil mix. We grew 24 seedlings with 20 cm spacing on each tank until harvest. A drip-irrigation system uniformly distributed the expected volume of water to all plants grown in the same treatment. To monitor the amount of rainfall during the experimental period, rainfall collectors were placed near the experimental site. The experiment was designed as a split-plot design in four replications with the two irrigation schemes as the main-plot factor: the sufficient- and restricted-irrigations, and the four stomatal model mutants and JHN control as the sub-plot factor in a randomized complete block (RCB). Each soil tank was considered an experimental unit. There were 40 experimental units (soil tanks) from two irrigations x five varieties x four replications.

Soil moisture and temperature were monitored daily at 12 cm below the soil surface from 3:00 p.m. using a soil moisture meter (Field Scout TDR150). From germination to maximum tillering, 5 L of water were irrigated daily to each experimental unit until the initiation of the R_1–2_ reproductive stage. The average total amount of water use during the vegetative stage was 340 L per soil tank. For sufficient irrigation, the amount of water needed to maintain 35–45% volumetric soil moisture content (VMC) is sufficient to maintain normal vegetative stage (5 L per day) and 10 L per day during the reproductive stage up to harvest. For restricted irrigation, 5 L per day during vegetative stage up to R_1–2_ stage and no additional irrigation was applied up to harvest. We encountered equivalent to 8 L of seasonal rainfall over the experimental site into the total water use. The total volumes of water use in the sufficient and restricted irrigations were 908 L and 348 L per soil tanks, respectively. The average VMC was 35–45 and 10% for sufficient and restricted irrigations, respectively. The average soil temperatures during the reproductive to harvest were 35–37 and 40–43°C for the sufficient and restricted irrigations, respectively. When the rice was approaching maturity, the number of panicles per cm^2^ and 1,000-seed weight were collected as defined:

a) % seed set = (seed-filled florets/total florets) × 100
b) Biomass is the dried weight of the whole above-ground plant
c) WUE is the dried weight of biomass (kg) per liter of water use.

### Statistical Analysis

Statistical analysis was undertaken based on the analysis of variance (ANOVA) to determine the main and interaction effects using the R version 3.4.3. Treatment means were compared using the least significant difference (LSD) to determine whether they were significantly different at the 0.05 probability level.

## Results

### Mutants With Altered Stomatal Density/Size

**FIGURE 1:**
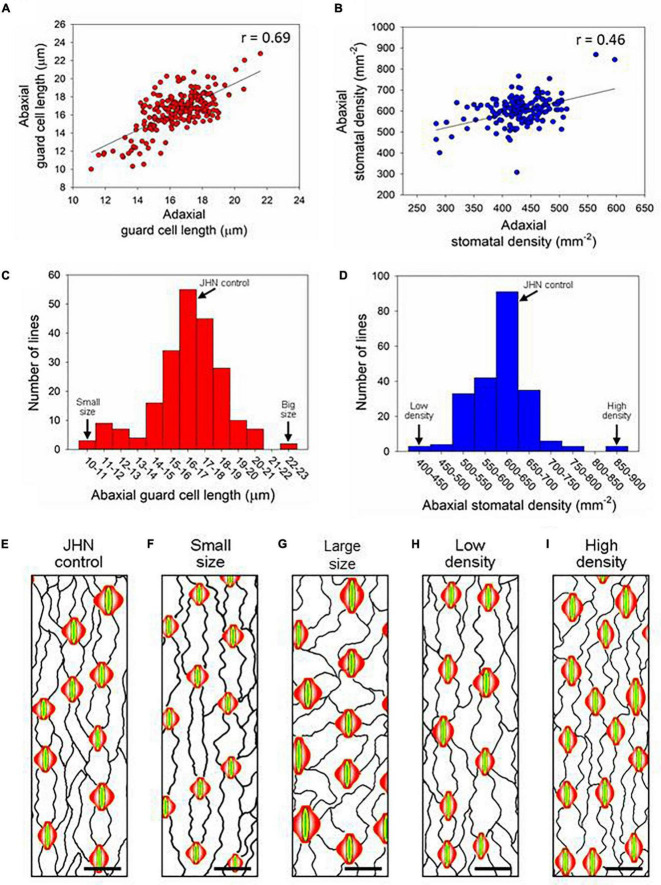
Stomatal size and density screening using the flag leaves of 216 M6 mutant core collection (MCC) members. XY scatter plots of abaxial and adaxial **(A)** stomatal size and **(B)** stomatal density. Distribution of the 216 M6 MCC lines according to **(C)** abaxial stomatal size and **(D)** abaxial stomatal density, where significant correlations were observed with Alpha.01 (99%). **(E–I)** Representative illustrative drawings based on tracings of the **(E)** Jao Hom Nin (JHN) wt and **(F–I)** lines identified as have **(F)** small (SS) or **(G)** Large stomata (LS), or **(H)** low (LD) or **(I)** high stomatal density (HD). Guard cells are indicated in yellow, and subsidiary cells, in red. The bin location of the four stomatal model mutant lines along with JHN are marked in **(C,D).** Scale bars = 25 μm.

**FIGURE 2:**
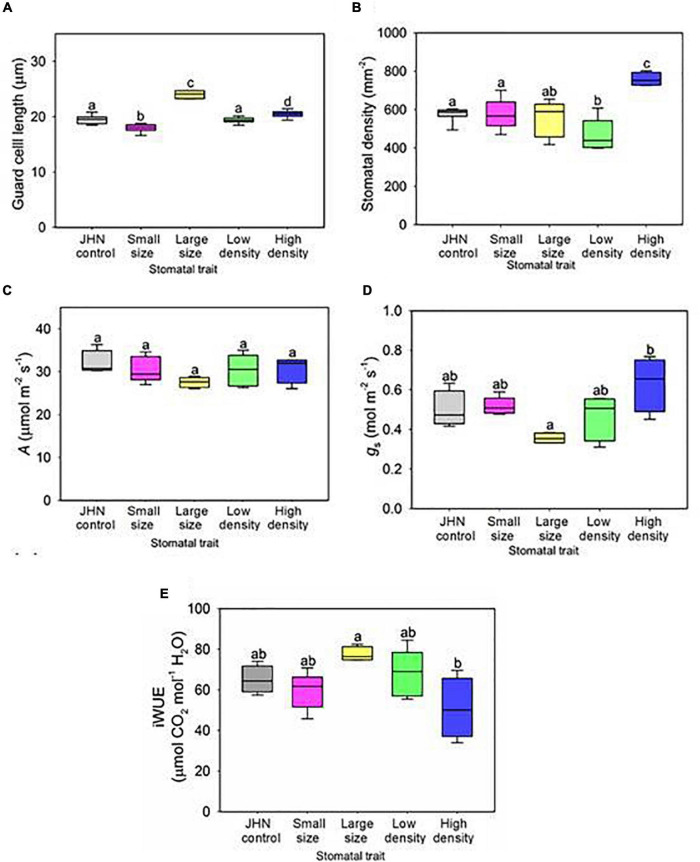
Gas exchange analysis of stomatal model mutants with altered density or size. Flag leaf **(A)** stomatal size and **(B)** density measurements conducted on all stomatal model mutants. **(C)** Carbon assimilation (*A*) and **(D)** stomatal conductance of stomatal model mutants under saturating light conditions (PAR 2,000 μmol m^–2^ s^–1^). **(E)** Improved water use efficiency (iWUE) of JHN and stomatal model mutants. Boxes indicate the upper (75%) and lower (25%) quartiles, and horizontal lines within the boxes indicate the median. Whiskers indicate the ranges of the minimum and maximum values, and different letters within a graph indicate a significant difference between the means of *p* < 0.05 (ANOVA, *post hoc* multiple comparisons, Holm–Sidak method). **(A,B)** *n* = 8; **(C–E)** *n* = 4–5.

Stomata can be detected on both the adaxial and abaxial surfaces of rice leaves aligned in rows with vertical leaf veins (Figure 1). Screening for flag-leaf stomatal density of 216 M_6_ MCC lines showed that 7.8 and 3.7% exhibited variations in stomatal density and size, respectively (Supplementary Table 1 and Table 1). Of the 216 M6 MCC members, six and eleven mutant lines exhibited higher and lower stomatal density than the JHN wild type, respectively. Five and three mutants exhibited bigger and smaller stomatal sizes than the JHN wild type, respectively. There were significant correlations between stomatal size on the adaxial and abaxial leaf surfaces (*r* = 0.69, α = 0.01) and stomatal density (*r* = 0.46, α = 0.01) on the adaxial and abaxial leaf surfaces. In addition, greater variability in stomatal density was observed on the adaxial (401—869 mm^–2^) than the abaxial (284–597 mm^–2^) leaf surfaces (Figure 1). The results also showed no correlation between the adaxial and abaxial leaf surfaces in terms of stomatal density and size (Supplementary Figure 1). We selected four stomatal model mutants that clearly exhibited SS, LS, LD, and HD to serve as a platform for identifying genes and to understand the physiological effect of these alterations on photosynthesis, WUE, and tolerance to drought (Figures 1C–L,2A,B, Supplementary Table 1, and Supplementary Figure 1). We evaluated the expression of stomatal density of flag leaves grown under different seasons and irrigation regimes. The results suggest that relative variation in stomatal density was more consistent in every measurement conducted in different seasons (Supplementary Table 6).

**TABLE 1:** Outcomes of the microscopic screening of stomatal density/size in the 216-member Mutant Core Collection (MCC).

Trait	Pop (*n*)	Selected mutants		Approach
		
		Increase	Decrease	
Stomatal density	Core (216)	6	11	No. of stomata per mm^2^
Stomatal size	Core (216)	5	3	Length of guard cell (cm)

Before physiological and drought experiments, the differences in stomatal traits of the model mutant lines were confirmed. Compared to the JHN control, both SS and LS retained significantly smaller or larger stomata, with the corresponding stomatal density of the SS and LS lines remaining comparable to the JHN control (ANOVA, *P* at least < 0.05) (Figures 2A,B). However, for the SS, the reduction in guard cell length (stomatal size) was not as large as when we isolated the line originally when grown under field conditions (see Figures 1C, 2A). Analysis of stomatal density in the LD and HD of mutant lines confirmed the phenotype detected in the original screen (ANOVA, *P* at least < 0.05) (Figures 1D, 2B). To ascertain whether the changes in guard cell length were also associated with the whole stomatal complex, we measured the total stomatal complex area in JHN controls and the four stomata mutant lines (Supplementary Figure 2). Interestingly, while most of the same trends were comparatively observed to guard cell length, the complex area of SS was not significantly different to JHN, implying that the changes that had arisen through FNB were specific to guard cell length and not to the whole stomatal complex. We also checked whether the stomatal phenotypes found on the flag leaf were present earlier during the vegetative growth stage but did not detect any significant differences between the FNB lines and the JHN control. However, the LS and HD line did have the highest values for size and density, respectively, compared to all the other lines (Supplementary Figure 3).

### Alteration of Stomatal Traits Affects Physiological Responses

We next wanted to understand how changes in stomatal size and density affect physiological responses, *A*, *g*_*s*_, and chlorophyll fluorescence (Fv/Fm). We found no significant differences in *A* or chlorophyll fluorescence (Fv/Fm) among the stomatal model mutants and JHN (Figure 2C and Supplementary Figure 4). We used IRGAs set to saturating light conditions (2,000 μmol.m^–2^ s^–1^ PAR) to measure the gas exchange of our plants and found no significant differences in *A* (Figure 2C), but the HD line did show a trend toward increased *g*_*s*_, with a significant difference detected between the HD and LS (Figure 2D). Given that HD plants also had relatively large stomata, this suggests that the increased stomatal density, rather than increased stomatal size, was probably the primary factor driving increased *g*_*s*_ in our HD line. Whilst none of the FNB lines displayed significantly different iWUE from the JHN control, the LS line did have higher iWUE than the HD plants, probably due to lower values of *g*_*s*_ (Figure 2E). In addition, stomatal model mutants are similar in agronomic traits, including days to flowering, leaf length, leaf width, tillers/plant, plant height, seed weight, and grain yield under non-restricted irrigation (Supplementary Tables 5, 6). Nonetheless, HD and LS were significantly different in *g*_*s*_ while LD, SS, and JHN were intermediate despite the apparent differences in stomatal density and size (Figure 2D and Supplementary Table 3).

### Response to Increased CO_2_

**FIGURE 3:**
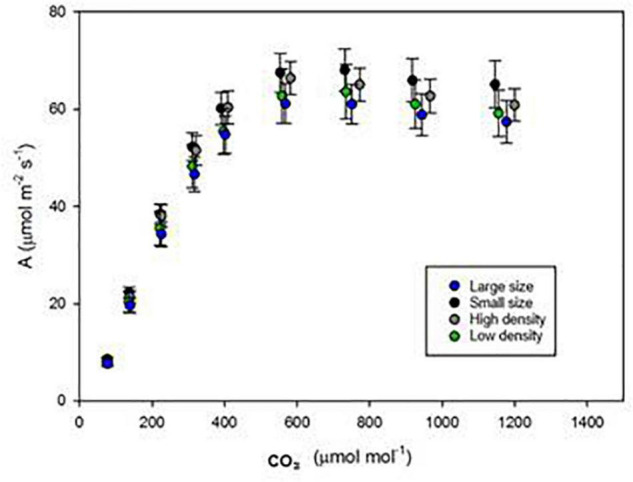
Assimilation (*A*) in response to changing carbon supplies of four mutant lines: JHN826 (Thind, 2019) JHN3117 (SS), JHN2447 (HD), and JHN8756 (LD). Error bars = S.E.M.

To understand if the alteration in the stomatal density or size affects the photosynthetic response to elevated [CO_2_], the flag-leaf scale photo-assimilation rates of stomatal model mutants were monitored from 100 to 1,500 ppm [CO_2_]. As the [CO_2_] was increased from 100 to 600 ppm, *A* gradually increased to maximized at 600 ppm and then slowly declined as the [CO_2_] reached 1,500 ppm (Figure 3). However, the photosynthetic responses (*A*) of all stomatal model mutants to elevated [CO_2_] were not significantly different and followed the same pattern. Despite HD and LS clearly showed significantly different *gs*, no advantage was observed in photosynthetic acclimation to elevated [CO_2_].

### Rapid Stomatal Response to Light-Dark-Light Condition

**FIGURE 4:**
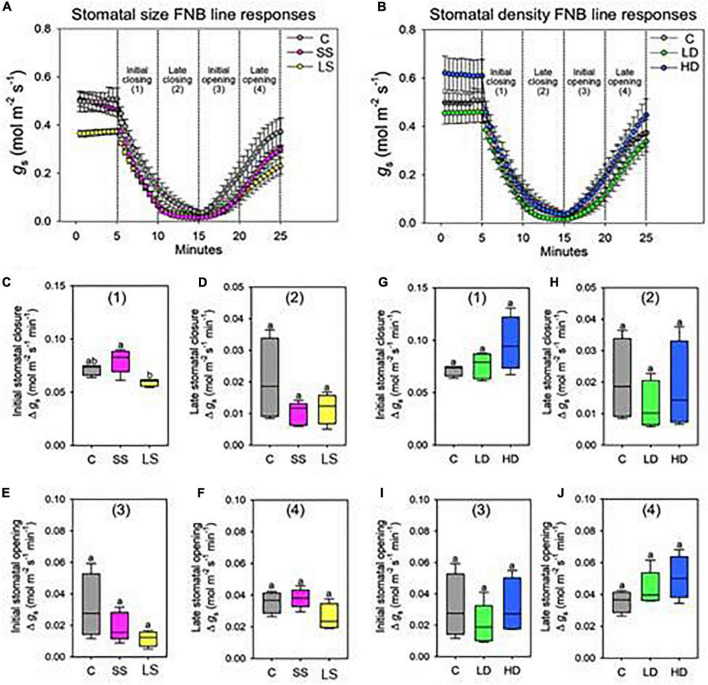
Stomatal size but not density significantly changes the speed of stomatal closure. Stomatal conductance (*g*_*s*_) responses to light-dark-light treatment over 20 min for **(A)** stomatal size mutants: small size (SS), large size (LS), and control (C). **(B)** Equivalent *g*_*s*_ response for stomatal density mutants: low density (LD), high density (HD), and C. Note, HD plants also have increased abaxial stomatal size relative to C, SS, and LD. Stomatal closure was instigated by 10 min of complete darkness (0 m^–2^ s^–1^ PAR) following “steady-state” conditions of saturating light (2,000 m^–2^ s^–1^ PAR). To re-open stomata saturating light was re-introduced. **(C–F)** Rate of Δ g_*s*_ per minute of stomatal size mutants analyzed in 5-min segments during **(C)** initial stomatal closure, **(D)** late stomatal closure, **(E)** initial stomatal opening, and **(F)** late stomatal opening. **(G–J)** Equivalent rate of Δ g_*s*_ per minute of stomatal density mutants during **(G)** initial stomatal closure, **(H)** late stomatal closure, **(I)** initial stomatal opening and **(J)** late stomatal opening. For graphs a and b error bars = S.E.M. For **(C–J)**, boxes indicate the upper (75%) and lower (25%) quartiles and horizontal lines within boxes indicate the median. Whiskers indicate the ranges of the minimum and maximum values, and different letters with a graph indicate a significant difference between the means to at least *p* < 0.05 (ANOVA, *post hoc* multiple comparisons, Holm-Sidak method). *n* = 4–5 plants.

We wanted to assess whether alterations in stomatal size or density in mutant line would impact on the rate (*g*_*s*_ mol m^–2^ min^–1^) of stomatal closure and subsequent re-opening in response to a rapid dark/light transition (Figure 4). The stomatal model mutants and JHN control were evaluated for rhythmic stomatal response by monitoring the changes in *g*_*s*_ in 10 min dark/light intervals. For each cycle, when the light was turned off, *g*_*s*_ slowly declined until 10 min, and when the light was turned on, *g*_*s*_ quickly continually increased up to the end of the 10 min illumination period. The responses of the FNB lines compared to the control are plotted in two separate groups, namely, FNB lines isolated during the stomatal size screen (Figures 4A,C–F) and FNB lines isolated during the stomatal density screen (Figures 4B,G–I). When comparing LD, HD, and JHN control, no significant differences in *g*_*s*_ were detected across any timelines, suggesting that variation in stomatal density does not have any effect on the rhythmic stomata response (Figures 4G–I). However, from the light-dark-light experiment to see how stomata response to light change, we found that SS was significantly faster than LS during the initial 5-min dark period only (ANOVA, *p* < 0.05) (Figure 4C). Beyond that point, no significant differences were detected, suggesting that stomatal size does not have a strong effect on rhythmic response to dark/light transitions (Figures 4C–F).

### Drought Response

**FIGURE 5:**
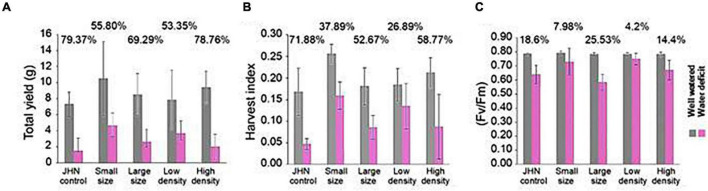
Yield performance of stomatal model mutants and JHN under conditions of sufficient water and 14 days withholding water: **(A)** total yield measured for both sufficient-water (gray) and drought conditions (pink), **(B)** harvest index for both sufficient-water (gray), and drought conditions (pink), and **(C)** F_*v*_/F_*m*_ measured 14 days after drought comparing sufficient-water (gray) and drought (pink) conditions. The numbers on top of the bar in the lower graphs indicate the reduction percentage. Statistical analysis was conducted using R statistical software.

Having measured various physiological parameters related to stomatal functioning, we next wanted to understand whether alterations in stomatal density and size had any impact on drought tolerance and WUE. We conducted a drought experiment by withholding water before R_1–2_ to create water stress around pollination. Under short-term drought conditions, water was withheld for 5 days just before the R_1–2_, the panicle initiation, followed by the 200 ml irrigation every other day up to 14 days. The results show that LD and SS produced higher quantum yield (F_v_/F_m_), grain yield, biomass (data not shown), and HI under the short-term restricted irrigation. When compared to the impact of the restricted and sufficient irrigations, LD and SS showed less reduction in F_v_/F_m_, grain yield, and HI than LS, HD, and JHN under the severe short-term drought (Figures 5A–C). These results suggest that LD and SS are less sensitive to short-term drought than HD, LS, and JHN.

To determine whether changes in stomatal density and size affect WUE, the stomatal model mutants were tested outdoors on large cement blocks to effectively control irrigation throughout the whole plant development cycle. The average VMC of the treatment with sufficient water and restricted water was 35–45 and 10%, respectively. The average soil temperatures of the sufficient water and restricted water treatments were 35–37 and 40–43°C, respectively, throughout the growing season. The total amount of water used with sufficient-water and restricted-water treatments was 908 and 348 L/CB, respectively. Based on the data collected, we found that restricted water greatly affected the percentage seed set, panicle/plant, biomass/plant, and WUE (Supplementary Tables 3, 4). Under the sufficient-water treatment, all stomatal model mutants showed similar WUE to JHN. In contrast, the restricted-water treatment strongly reduced percentage seed set, panicle/plant, biomass/plant, and WUE in HD (Table 2). LD performed better than HD in all agronomic traits and showed the highest biomass and WUE of all stomatal model mutants studied. Since LD effectively maintained quantum yield, HI, biomass, grain yield, and WUE under such long-term drought conditions, the results suggest that rice breeders should pay more attention to reducing stomatal density in developing new WUE rice varieties to save at least 50% of irrigation water and improve drought tolerance of rice.

**TABLE 2:** The % seed set, panicle/plant, biomass/plant, and water-used efficiency (WUE) among stomatal model mutants and Jao Hom Nin (JHN) grown in sufficient-water and restricted-water conditions.

Treatment	Lines	% seed set		Panicle/plant		Biomass/plant		WUE g/L	
Sufficient water	JHN	84.50	a	16.23	ab	40.81	ab	0.61	e
Sufficient water	BS	84.20	a	17.43	a	39.81	abc	0.60	e
Sufficient water	HD	71.48	c	12.00	cd	40.31	abc	0.61	e
Sufficient water	SS	86.70	a	15.65	b	37.91	c	0.57	e
Sufficient-water	LD	79.18	b	15.58	b	38.36	bc	0.58	e
Restricted water	JHN	35.60	f	5.60	g	20.51	fg	0.80	b
Restricted water	BS	35.55	f	5.75	fg	18.55	gh	0.73	bc
Restricted water	HD	28.15	g	6.68	ef	17.50	h	0.69	cd
Restricted water	SS	39.53	def	7.65	e	20.13	g	0.79	b
Restricted water	LD	36.48	ef	7.50	e	23.69	de	0.93	a
Mean									
Sufficient water		81.12	a	15.38	a	39.38	a	0.59	b
Restricted water		35.60	B	6.64	B	20.08	B	0.79	a
*F*-test									
Water conditions		**		**		**		**	
Line		**		**		**		**	
Water condition × Line		**		**		**		**	

Different letters denoted statistical difference at p ≤ 0.05. ** indicates statistically difference at p ≤ 0.01.

## Discussion

### Generating Rice With Stomatal Variation *via* Induced Mutation and Genetically Modified Technologies

In the current study, we have obtained promising rice mutants with altered stomatal traits with fast-neutron-induced mutagenesis in JHN to help breeders successfully exploit and understand the value of new genetic variations to improve WUE, which is not possible with the current narrow genetic-based rice breeding. The stomatal model mutants, with distinct stomatal density and size, on an otherwise similar genetic background, provide the basis for linking the effects of stomatal traits on abiotic stress responses.

Despite the relatively significant differences in density and size of stomata found on flag leaves of the stomatal model mutants, environmental conditions influence the expression of stomatal density during vegetative growth. Unlike GM plants generated by the modification of orthologs of EPF, the expression of stomatal density was more consistent throughout plant development (Hughes et al., 2017; Caine et al., 2018). As per the recent developments, reduced stomatal density in rice can be developed by two transgenic approaches using orthologs of EPF genes. One approach is through overexpression of the EPF1, a negative regulator of stomatal density, resulting in rice and barley with markedly reduced stomatal density and improved iWUE (Hughes et al., 2017; Caine et al., 2018). The stomatal density of transgenic IR64OsEPF1 was 51–74% lower than that of the IR64 wild type (Caine et al., 2018). Another approach based on knockout EPFL9 expression, a positive regulator of stomatal density, using the Crisper-Cas9/CpF1 also generated IR64 with an 8-fold reduction in stomatal density (Yin et al., 2017). In the current study, using the fast-neutron-induced mutagenesis in JHN rice resulted in an 18.8 and 38.5% reduction in stomatal density from JHN wild type and the high-density mutant (HD), respectively. A more minor reduction in stomatal density suggests that the genetic regulation of altered stomatal traits induced by mutation and genetic engineering does not arise from the same genetic factors (Franks and Beerling, 2009; Doheny-Adams et al., 2012).

Despite the promising potential of CRISPR for future plant breeding, the technique has not yet been fully approved in many countries. One of the CRISPR techniques, the site-directed nuclease-1 (SDN-1), has been recently approved as a non-GMO product in several countries such as Argentina, Brazil, Chile, Columbia, China, Japan, and Australia (Zhang et al., 2020). However, different countries have different regulations, and some of them, including Thailand, still oppose GM crops.

### Smaller Stomata Respond More Rapidly to Changing Light Intensity

Recent work on a range of plant species has shown that the small dumbbell-shaped stomata of grasses respond rapidly to the changing light intensities (McAusland et al., 2016; Lawson and Vialet-Chabrand, 2018). The pace at which stomata can react to the changing environment is essential for improving short-term WUE (McAusland et al., 2016; Lawson and Vialet-Chabrand, 2018). Plants with SS, and in some cases, HD has been shown to have a higher dynamic range of *g*_*s*_, which allows leaves to adapt more quickly to changing environments, such as temporary shading (Ohsumi et al., 2007; Drake et al., 2013; McAusland et al., 2016; Lawson and Vialet-Chabrand, 2018). In our stomatal model mutants, their density and size on the leaf surface did not affect the speed of stomatal aperture. Instead, smaller stomata were significantly faster than the larger stomata during the initial closure. It would be interesting to test whether smaller stomata can elicit a similar response under low light intensity or shading.

### Rice With Lower Stomatal Density Is More Water-Use Efficient Under Drought Conditions

Availability of irrigation water will be a significant constraint for rice productivity in the imminent climate change. Rice consumes 34–43% of total world irrigation water. Around 1.3–1.4 tons of water is required for rice production in Asia (How to Manage Water, IRRI Knowledge Bank); thus, improving WUE and water productivity have been major goals for water-saving and improved biomass production in irrigated and water-limited areas (Kijne et al., 2003; Passioura, 2006). Moreover, selection for high WUE *per se* without improving photosynthetic capacity has led to reduced transpiration, crop water use, plant biomass production, and low grain yield (Blum, 2009; Flexas et al., 2016). Our study results, using stomatal model mutants, demonstrated that rice mutants with low stomatal density (LD) do not necessarily lead to low productivity. Comparison among stomatal model mutants under long-term water stresses revealed that LD maintained the highest WUE and the highest biomass/plant, panicle/plant, and% seed set, albeit similar WUE among stomatal model mutants and JHN wild type under adequate water availability. In addition, short-term water stress did not affect maximum quantum yield (Fv/Fm), harvest index (HI), and grain yield (GY) of LD. Thus, LD may have better physiological responses to protect functional photosystem II under water stress conditions. Results from IR64-OsEPFoe lines support the findings that lower stomatal density reduces water use by 60% under drought conditions during the seedling stage (Caine et al., 2018). Moreover, these LD GM plants were cooler under drought, but not under well-watered conditions (Caine et al., 2018). These results may indicate that under drought stress, LD plants may conserve higher leaf water content and mitigate the effects of heat stresses as a consequence. One of the significant difficulties in using WUE as a primary selection criterion for rice breeding is how to precisely assess field WUE for many genotypes under different drought conditions. We propose that low stomatal density can enabling plant breeders to design a robust screening of a large number of germplasms, followed by field biomass and grain yield testing of selected lines for precisely improving WUE in rice.

## Conclusion

In this study, we demonstrated the genetic potential of fast-neutron mutagenesis to rapidly induce genetic variation. By using 216 mutant populations generated from JHN variety, we have screened several stomata traits that thought can be useful for plant phenotype improvement. As stomata were known in functioning as gates for gas exchanges, here, we have found a non-transgenic mutant line with smaller or larger stomatal size, or lower or higher stomatal density, to evaluate the impact of altered stomatal density and size on the responses to abiotic stresses and water use efficiency. The stomatal model mutants share a similar *A*, and the line with HD had the highest stomatal conductance. Small stomata respond faster to dark/light cycles and better to high CO_2_ concentration gradients above 600 ppm. In the drought conditions, SS and LD were associated with less reduction in grain yield, HI, and F_*v*_/F_*m*_. LD consistently performed well in the field, especially in improving biomass production and WUE. Therefore, reducing stomatal density will empower plant breeders to effectively develop climate-ready rice, thus lessening the impact of drought stress and the imminent threat of climate change.

## Data Availability Statement

The original contributions presented in the study are included in the article/Supplementary Material, further inquiries can be directed to the corresponding author/s.

## Author Contributions

AV, JG, and SA conceived the concept of the experiment and the manuscript. MP, RC, CH, EH, JS, CC, CP, and RN performed the experiments. MP, RC, and AV wrote the manuscript and analyzed the data. SR maintained the JHN mutant population. AV revised the manuscript. All authors reviewed and approved this submission.

## Conflict of Interest

The authors declare that the research was conducted in the absence of any commercial or financial relationships that could be construed as a potential conflict of interest.

## Publisher’s Note

All claims expressed in this article are solely those of the authors and do not necessarily represent those of their affiliated organizations, or those of the publisher, the editors and the reviewers. Any product that may be evaluated in this article, or claim that may be made by its manufacturer, is not guaranteed or endorsed by the publisher.
