# Supplementary material for: Induced Genetic Variations in Stomatal Density and Size of Rice Strongly Affects Water Use Efficiency and Responses to Drought Stresses
Source: Front Plant Sci. 2022 May 25;13:801706. doi: 10.3389/fpls.2022.801706 (PMC9174926; doi:10.3389/fpls.2022.801706)
Supplement: Supplementary file 1 [file Data_Sheet_1.pdf]

SUPPLEMENTARY

Supplementary Table 1. Stomata Size and Stomata density measurement on 206 Mutant core collection (Excel file)**.**

Supplementary Table 2. Stomata Size and Stomata density measurement conducted on JHN and four selected mutant line before drought experiment respectively

Treatments		stomata density (mm^2)		stomata size (mm)	
**Irrigation conditions**					
Sufficient		571		14.15	
Restricted		579		13.56	
	F-test	ns		ns	
**Lines**					
Control JHN		626	ab	13.69	bcd
Large Size		604	bc	15.18	a
High Density		584	cd	13.44	cd
Small Size		639	a	13.12	d
Low Density		541	ef	14.34	b
	F-test	**		**	
**Irrigations x Lines**					
	F-test	ns		ns	
CV a (%)		3		3.90	
CV b (%)		4		5.10	
Mean		575		13.86	

Supplementary Table 3. Water applied from germination to harvest and the amount of rainfall during the experiment between Sufficient-water and Restricted-water conditions (Liter per cement block, CB**)

Growth Stages	Water Use(liter/day/area(	Sufficient-water	Restricted-water
Vegetative stage	5	340	340
Reproductive stage	10	560	0
Rainfall	8	8	8
Total water used		908	348

Supplementary Table 4. Leaf rolling, leaf drying, maximum quantum yield (Fv/Fm) and percentage of recovery of JHN mutant lines with various stomata types under long term drought condition (n=3)

Treatment	Varieties	7 days after treatment			14 day after treatment			%Recovery
		Leaf rolling	Leaf drying	Fv/Fm	Leaf rolling	Leaf drying	Fv/Fm	
Sufficient Irrigation	JHN	1.0	0	0.800	1.0	0	0.787	100
	LD	1.3	0	0.797	1.3	0	0.783	100
	HD	1.0	0	0.797	1.0	0	0.783	100
	SS	1.0	0	0.807	1.0	0	0.793	100
	LS	1.3	0	0.797	1.7	0	0.783	100
	**Mean**	**1.1**	**0**	**0.800**	**1.2**	**0**	**0.786**	**100**
Restricted Irrigaton	JHN	4.3	38.3	0.690	4.7	56.7	0.640	60
	LD	3.3	15.0	0.783	3.3	23.3	0.750	80
	HD	3.7	40.0	0.710	4.7	60.0	0.670	60
	SS	4.3	16.7	0.767	4.7	35.0	0.730	73
	LS	4.3	63.3	0.620	5.0	76.7	0.583	60
	**Mean**	**4.0**	**34.7**	**0.714**	**4.5**	**50.3**	**0.675**	**66**
	$LSD_{0.05}$							
	TRT	0.74	2.78	0.063	0.87	11.82	0.031	3.65
	VAR	ns	13.87	ns	ns	17.33	0.064	ns
	TRT*VAR	ns	19.62	ns	ns	24.50	ns	ns

Supplementary Table 5. Pearson correlation coefficient between each pair of variables for stomatal, leaf, and agronomic traits

	AB-D (mm2)	*AD-D (mm2)*	*50% Flowering*	*Maturity*	*Pl Height*	*Tillers/Pl*	*1000 S_wt*	*Leaf length (cm)*	*Leaf width (cm)*	*Grain Yield (kg./ha)*
**AB-D (mm2)**	**1.00**	***0.5241******	**-0.2524**	**-0.1684**	**0.0417**	**-0.1543**	**0.0944**	**-0.1438**	**-0.0367**	**0.0442**
**AD-D (mm2)**		**1.00**	**0.0046**	**-0.0157**	**0.0619**	**-0.1179**	**0.0453**	**-0.1346**	**-0.0406**	**0.1412**
**50%Flowering**			**1.00**	***0.7707******	**-0.1082**	**-0.2610**	***-0.4049******	**0.2381**	**0.2127**	**0.1822**
**Maturity**				**1.00**	**-0.1795**	**-0.1253**	***-0.3143*****	***0.3342*****	**0.1939**	**0.1939**
**Pl Height**					**1.00**	**0.1979**	***0.5219******	**0.1644**	**0.2156**	**0.0347**
**Tillers/Pl**						**1.00**	**0.0724**	**0.0029**	**-0.1991**	**-0.0599**
**1000 S_wt**							**1.00**	**0.0596**	**0.2469**	**0.1640**
**leaf length (cm)**								**1.00**	**0.2842**	***0.6328******
**leaf wide (cm)**									**1.00**	***0.3554*****
**Yield (kg./ha)**										**1.00**

AB-D: Abaxial density
AD-D: Adaxial density

The StatAdvisor
This table shows Pearson product moment correlations between each pair of variables. These correlation coefficients range between -1 and +1 and measure the strength of the linear relationship between the variables. Also shown in parentheses is the number of pairs of data values used to compute each coefficient. The third number in each location of the table is a P-value which tests the statistical significance of the estimated correlations. P-values below .05 indicate statistically significant non-zero correlations at the 95.0% confidence level. The following pairs of variables have P-values below .05:

AB-D (mm2) and AD-D (mm2)
50%Flowering and Maturity
50%Flowering and 1000 S_wt
Maturity and 1000 S_wt
Maturity and leaf length (cm)
Pl Height and 1000 S_wt
leaf length (cm) and Yield (kg./ha)
leaf wide (cm) and Yield (kg./ha)

Supplementary Table 6. Growth, yield, biomass and reduction in yield of JHN mutant lines with various stomata types under long term drought condition condition (n=3)

Treatment	Varieties	height (cm)	tiller number	flowering date	100 seed (g)	yield (g/plant)	shoot dry weight (g/plant)	harvest index	grain yield reduction
Sufficient Irrigation	JHN	76.7	14.3	98.3	1.81	7.31	42.85	0.168	0
	LD	71.7	11.7	99.0	1.95	7.85	42.24	0.185	0
	HD	78.3	10.7	96.7	2.02	9.40	43.85	0.213	0
	SS	74.3	10.0	98.7	2.14	10.48	41.17	0.256	0
	LS	77.7	15.3	97.7	2.02	8.51	47.47	0.181	0
	**Mean**	**75.7**	**12.4**	**98.1**	**2.00**	**8.71**	**43.52**	**0.201**	**0**
Restricted irrigation	JHN	75.0	14.7	102.7	1.57	1.51	32.70	0.047	76.5
	LD	74.0	12.3	103.0	1.75	3.66	30.54	0.135	47.9
	HD	70.7	14.7	102.3	1.81	2.00	25.26	0.088	79.7
	SS	72.0	10.0	102.0	1.90	4.63	28.75	0.159	54.2
	LS	78.0	11.3	103.0	1.84	2.61	30.94	0.086	66.3
	**Mean**	**73.9**	**12.6**	**102.6**	**1.774**	**2.88**	**29.64**	**0.103**	**64.9**
	$LSD_{0.05}$								
	TRT	ns	ns	0.58	0.093	2.78	8.78	0.052	2.12
	VAR	ns	ns	ns	0.156	2.10	ns	0.049	ns
	TRT*VAR	ns	ns	ns	ns	ns	ns	ns	ns

SUPPLEMENTARY FIGURES

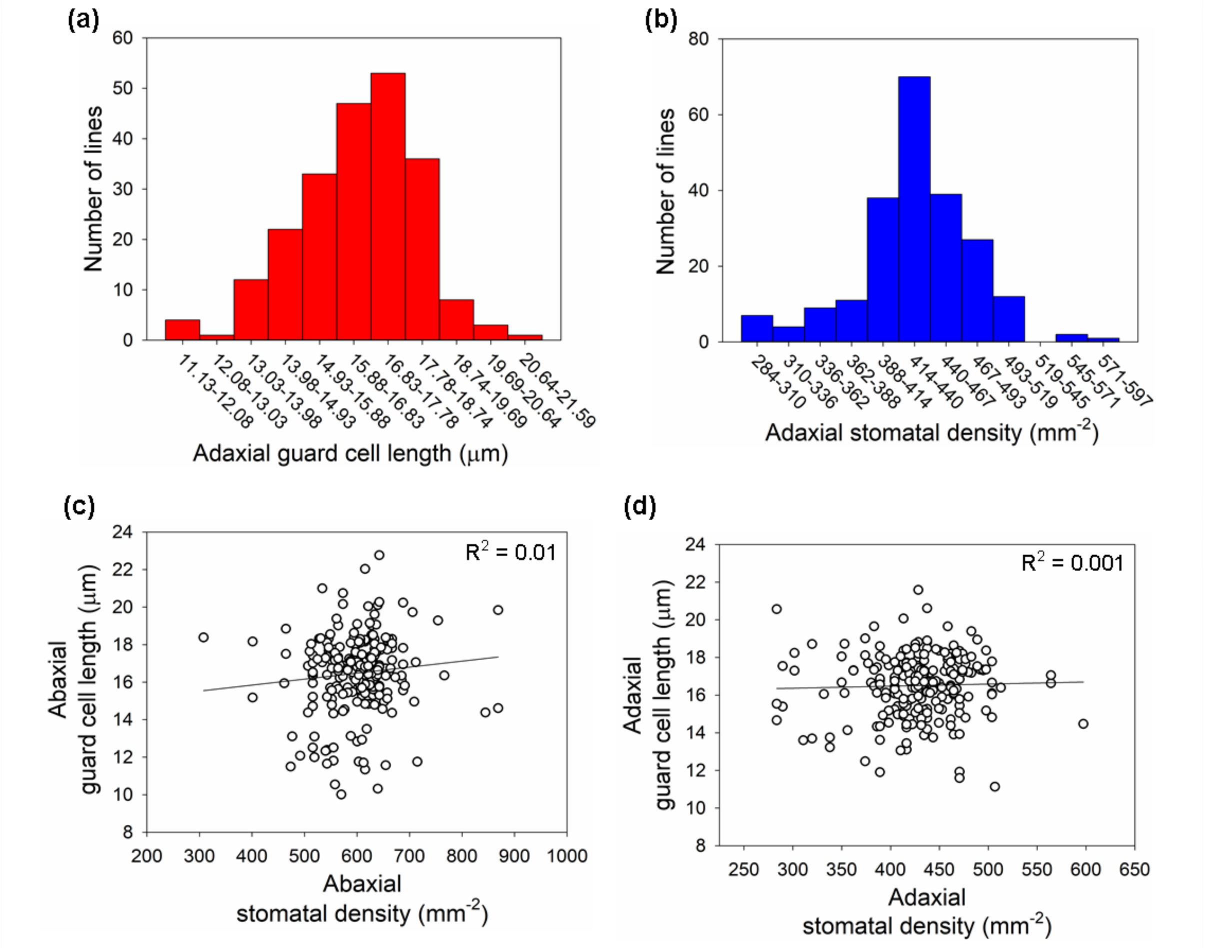

Supplementary Figure 1. Flag leaf stomatal size and density variation generated by Fast Neutron Bombardment. Distribution of 220 plants measured during phenotypic screening for (**a**) adaxial Guard cell length (size) and (**b**) stomatal density. X-Y scatter plots of (**a**) abaxial stomatal size and density and (**d**) adaxial stomatal size and density. No correlation was found from both scatter plot.

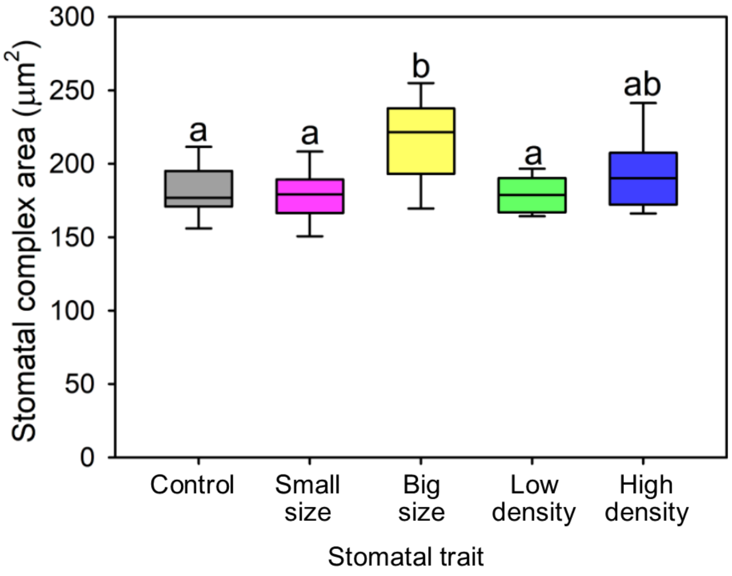

Supplementary Figure 2. Stomatal complex area of JHN control plants and Fast Neutron Bombarded lines with different stomatal size (S) and or density (D). Boxes indicate the upper (75%) and lower (25%) quartiles and horizontal lines within boxes indicate the median. Whiskers indicate the ranges of the minimum and maximum values, and different letters indicate a significant difference between means to at least $P < 0.05$ (ANOVA, post-hoc multiple comparisons, Holm-Sidak method). n = 8 plants.

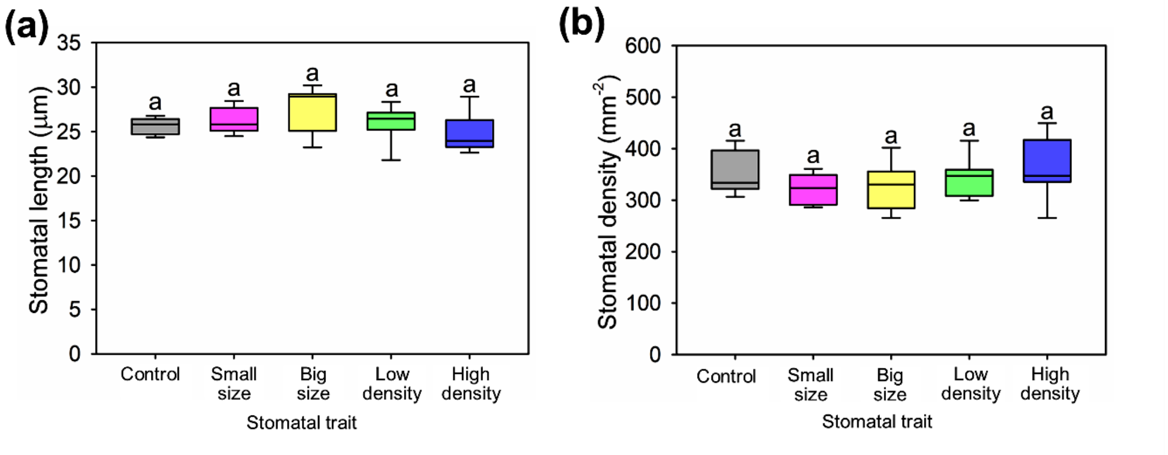

Supplementary Figure 3. Analaysis of Stomatal traits on 5 week-old plants. (**a**) Stomatal length (size) and (**b**) stomatal density measured on the largest fully expanded leaves of JHN controls, Small and Large size stomata and Low and High density stomata. Boxes indicate the upper (75%) and lower (25%) quartiles and horizontal lines within boxes indicate the median. Whiskers indicate the ranges of the minimum and maximum values. No significant different letters were detected between plants (ANOVA, post-hoc multiple comparisons, Holm-Sidak method). n = 8 plants.

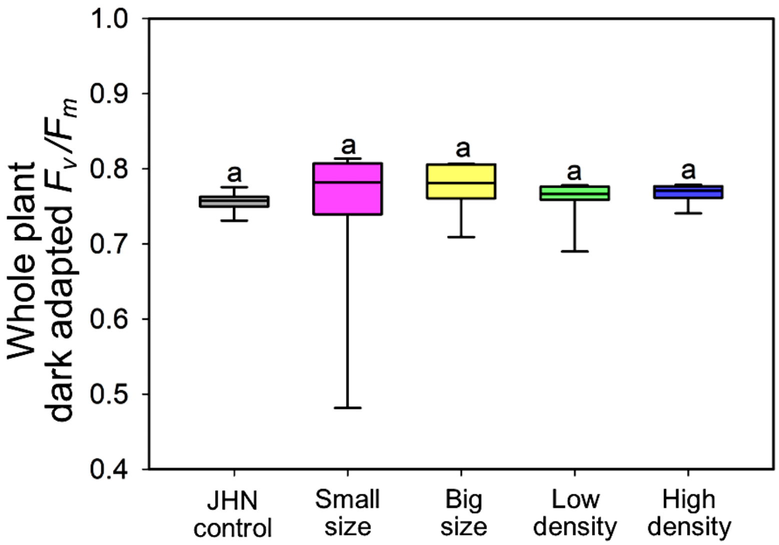

Supplementary Figure 4. Whole plant fluorescence F_v/F_m measurements of JHN wt and the four stomatal model mutants with different stomatal size (S) and or density (D) measured upon the emergence of flowers. Boxes indicate the upper (75%) and lower (25%) quartiles and horizontal lines within boxes indicate the median. Whiskers indicate the ranges of the minimum and maximum values. No significant different letters were detected between plants (ANOVA, post-hoc multiple comparisons, Holm-Sidak method). n = 8 plants.
