# Supplementary material for: Induced Genetic Variations in Stomatal Density and Size of Rice Strongly Affects Water Use Efficiency and Responses to Drought Stresses
Source: Front Plant Sci. 2022 May 25;13:801706. doi: 10.3389/fpls.2022.801706 (PMC9174926; doi:10.3389/fpls.2022.801706)
Supplement: Supplementary file 2 [file Data_Sheet_3.PDF]

No	Field Code	Mutant Name	Abaxial Size	Adaxial size	Abaxial density (mm²)	Adaxial density (mm²)
1	1	Mu361	18.50	18.11	346.67	206.67
2	2	Mu481	20.23	18.34	380.00	260.00
3	3	Mu370	19.84	16.63	480.00	311.67
4	4	Mu520	20.10	18.71	353.33	176.67
5	6	Mu623	20.04	16.84	343.33	260.00
6	7	Mu724	15.30	18.05	348.33	193.33
7	8	Mu822	20.27	15.80	355.00	238.33
8	***9***	***Mu826***	***22.77***	***21.59***	***363.33***	***211.67***
9	11	Mu843	18.23	19.65	355.00	236.67
10	12	Mu953	19.10	17.58	331.67	220.00
11	13	Mu1039	21.00	20.07	295.00	228.33
12	14	Mu1069	15.44	15.44	363.33	260.00
13	15	Mu1086	17.31	18.19	340.00	236.67
14	16	Mu1082	22.04	20.61	340.00	241.67
15	17	Mu1096	16.61	15.78	361.67	253.33
16	18	Mu1444	18.20	19.65	338.33	253.33
17	19	Mu1452	15.95	18.64	285.00	256.67
18	20	Mu1640	17.16	18.82	305.00	241.67
19	21	Mu1644	16.77	17.57	318.33	255.00
20	22	Mu2290	16.38	18.07	300.00	215.00
21	23	Mu1742	18.16	15.39	221.67	160.00
22	24	Mu1747	19.01	15.07	311.67	241.67
23	25	Mu1819	17.81	16.03	368.33	256.67
24	26	Mu1830	16.88	14.33	316.67	213.33
25	27	Mu1895	19.61	15.41	350.00	225.00
26	28	Mu1911	18.16	15.27	340.00	241.67
27	29	Mu1913	15.70	18.95	303.33	270.00
28	30	Mu1924	16.86	17.70	280.00	228.33
29	33	Mu1974	16.74	15.79	305.00	260.00
30	34	Mu2119	17.50	15.53	256.67	156.67
31	39	Mu2808	16.70	17.90	330.00	218.33
32	40	Mu3217	18.39	18.19	328.33	261.67
33	42	Mu3234	17.74	18.72	308.33	195.00
34	43	Mu3258	18.32	18.24	301.67	166.67

35	44	Mu3446	17.22	17.32	346.67	200.00
36	46	Mu4201	20.74	17.47	316.67	230.00
37	48	Mu4856	14.71	16.08	285.00	223.33
38	49	Mu4851	16.42	17.74	316.67	230.00
39	51	Mu4873	17.02	16.44	285.00	223.33
40	52	Mu4903	18.30	17.67	368.33	235.00
41	53	Mu4906	18.29	17.36	350.00	271.67
42	54	Mu4943	19.73	16.52	390.00	255.00
43	55	Mu4974	16.05	18.37	350.00	275.00
44	56	Mu4978	16.53	16.64	365.00	236.67
45	57	Mu5017	14.96	14.82	360.00	278.33
46	58	Mu5018	15.47	15.21	353.33	240.00
47	59	Mu5019	15.29	17.18	336.67	266.67
48	60	Mu5100	12.39	13.87	300.00	240.00
49	61	Mu5119	15.56	15.90	333.33	250.00
50	62	Mu5126	17.24	17.28	323.33	251.67
51	66	Mu5144	17.06	16.34	393.33	236.67
52	67	Mu5147	16.11	16.66	378.33	278.33
53	71	Mu5281	16.94	18.15	380.00	258.33
54	72	Mu5308	17.76	17.31	350.00	273.33
55	73	Mu5332	17.33	18.19	338.33	255.00
56	74	Mu5340	17.73	16.10	356.67	278.33
57	76	Mu5367	16.90	15.24	355.00	223.33
58	79	Mu5390	17.67	14.53	301.67	248.33
59	84	Mu2409	18.31	18.37	291.67	228.33
60	85	Mu5504	16.12	16.20	333.33	241.67
61	87	Mu5569	16.60	16.76	351.67	215.00
62	89	Mu5571	17.27	18.59	348.33	238.33
63	92	Mu5574	17.20	18.05	341.67	226.67
64	93	Mu5593	19.03	17.75	346.67	223.33
65	94	Mu5633	16.23	17.69	313.33	208.33
66	96	Mu977	19.28	19.39	416.67	266.67
67	102	Mu1667	15.78	16.05	310.00	183.33
68	103	Mu1663	19.06	18.36	351.67	248.33
69	105	Mu1853	18.04	16.05	350.00	225.00
70	107	Mu4	18.25	16.66	340.00	210.00

71	109	Mu1306	17.82	17.89	380.00	253.33
72	110	Mu1309	11.81	11.93	306.67	260.00
73	111	Mu1781	18.30	17.67	365.00	233.33
74	112	Mu2313	18.29	17.36	335.00	273.33
75	113	Mu1818	13.11	13.60	263.33	171.67
76	114	Mu1822	17.35	17.22	345.00	265.00
77	115	Mu1255	17.57	18.01	351.67	236.67
78	117	Mu2550	18.62	17.41	328.33	256.67
79	120	Mu4053	16.23	19.01	346.67	206.67
80	121	Mu4539	15.56	15.05	380.00	260.00
81	123	Mu4814	14.61	17.05	480.00	311.67
82	124	Mu4821	10.32	13.69	353.33	176.67
83	125	Mu4840	14.84	15.05	343.33	260.00
84	126	Mu4906	15.61	16.67	348.33	193.33
85	127	Mu4913	15.30	14.43	355.00	238.33
86	128	Mu4932	15.12	16.47	363.33	211.67
87	129	Mu4943	15.81	15.05	355.00	236.67
88	131	Mu5547	12.83	14.54	331.67	220.00
89	132	Mu5678	17.05	15.53	295.00	228.33
90	134	Mu5863	17.73	17.90	363.33	260.00
91	136	Mu6158	16.38	18.19	340.00	236.67
92	137	Mu6192	15.40	18.49	340.00	241.67
93	139	Mu6693	16.73	17.57	361.67	253.33
94	140	Mu6712	16.76	18.07	338.33	253.33
95	141	Mu8097	15.21	16.11	285.00	256.67
96	142	Mu8419	15.10	16.20	305.00	241.67
97	143	Mu8714	14.55	14.71	318.33	255.00
98	144	Mu8754	18.10	18.60	300.00	215.00
99	145	***Mu8756***	***15.18***	***17.54***	***221.67***	***160.00***
100	148	Mu9396	17.33	14.19	311.67	241.67
101	150	Mu10599	14.36	13.90	368.33	256.67
102	151	Mu10699	14.45	16.12	316.67	213.33
103	152	Mu10965	14.79	14.97	350.00	225.00
104	153	Mu10997	14.84	14.78	340.00	241.67
105	154	Mu11152	18.55	17.53	303.33	270.00
106	158	Mu11582	14.38	14.89	280.00	228.33

107	159	Mu11930	14.79	13.92	305.00	260.00
108	160	Mu11940	18.84	20.56	256.67	156.67
109	161	Mu7-8-3	17.94	16.12	330.00	218.33
110	164	Mu6138	17.85	17.30	301.67	166.67
111	165	Mu6158	17.06	17.30	346.67	200.00
112	166	Mu4913	16.68	17.70	316.67	230.00
113	168	Mu1463	17.43	16.31	285.00	223.33
114	169	Mu1463	16.74	15.70	316.67	230.00
115	170	Mu2404	16.36	16.86	285.00	223.33
116	171	Mu2409	15.71	14.74	321.67	231.67
117	172	Mu2429	15.75	15.07	353.33	235.00
118	173	Mu2434	17.87	17.44	313.33	236.67
119	***174***	***Mu2447***	***14.38***	***14.47***	***466.67***	***330.00***
120	175	Mu2456	16.90	14.76	313.33	238.33
121	176	Mu2474	16.71	17.47	325.00	213.33
122	177	Mu2491	15.74	17.33	348.33	238.33
123	178	Mu2495	17.08	16.95	326.67	223.33
124	179	Mu2518	16.30	16.64	351.67	233.33
125	180	Mu2537	12.33	14.66	298.33	156.67
126	181	Mu2538	16.31	16.64	316.67	248.33
127	182	Mu2529	16.60	15.06	311.67	241.67
128	183	Mu2531	11.34	13.10	340.00	230.00
129	184	Mu2532	18.04	17.66	330.00	263.33
130	185	Mu2546	17.85	17.36	356.67	275.00
131	186	Mu2540	16.74	17.02	366.67	228.33
132	187	Mu2544	11.76	13.44	333.33	230.00
133	188	Mu2559	13.10	14.28	286.67	230.00
134	189	Mu2566	18.10	17.83	335.00	240.00
135	190	Mu2573	17.40	17.13	346.67	231.67
136	191	Mu2581	12.08	13.61	271.67	215.00
137	192	Mu2604	18.09	16.88	325.00	238.33
138	193	Mu1818	17.41	17.90	350.00	263.33
139	194	Mu3035	16.40	16.40	353.33	250.00
140	195	Mu3050	11.65	11.90	300.00	215.00
141	196	Mu3075	18.37	18.04	361.67	266.67
142	197	Mu3078	19.38	18.82	310.00	236.67

143	199	Mu3099	17.37	17.31	300.00	240.00
144	201	Mu3109	16.52	18.71	326.67	235.00
145	202	Mu3113	18.38	18.51	170.00	235.00
146	***203***	***Mu3117***	***10.01***	***11.13***	***315.00***	***280.00***
147	204	Mu3125	17.60	17.34	330.00	211.67
148	205	Mu3128	16.45	18.90	346.67	241.67
149	206	Mu3130	17.64	17.71	281.67	233.33
150	207	Mu3137	15.85	17.20	345.00	255.00
151	208	Mu3133	18.51	17.33	348.33	261.67
152	209	Mu3158	11.50	13.06	261.67	226.67
153	210	Mu3166	11.76	15.43	395.00	230.00
154	211	Mu3191	15.63	16.16	311.67	241.67
155	212	Mu3224	14.32	15.18	306.67	220.00
156	213	Mu4177	10.54	14.38	308.33	255.00
157	214	Mu4187	15.63	15.71	331.67	230.00
158	215	Mu4213	12.00	12.48	286.67	206.67
159	216	Mu4295	16.49	16.39	285.00	233.33
160	218	Mu4471	17.58	16.74	350.00	231.67
161	219	Mu4482	17.47	16.75	336.67	223.33
162	220	MuFRO	15.81	17.27	381.67	255.00
163	221	Mulox	12.51	13.75	306.67	245.00
164	222	MuMT1	14.95	16.38	331.67	235.00
165	223	MuMT2	12.93	15.73	336.67	231.67
166	226	Mu12854	15.47	15.81	326.67	233.33
167	227	Mu13328	12.51	13.75	285.00	186.67
168	228	Mu13353	17.55	15.85	308.33	218.33
169	229	Mu13471	16.74	15.18	335.00	275.00
170	230	Mu13337	14.95	14.40	391.67	238.33
171	233	Mu-X1	13.50	13.23	341.67	186.67
172	234	Mu4	16.38	18.44	333.33	246.67
173	235	Mu316	16.42	17.19	343.33	243.33
174	236	Mu361	17.25	15.24	311.67	250.00
175	239	Mu-X2	11.57	11.59	361.67	260.00
176	240	Mu1819	16.81	18.25	331.67	258.33
177	241	Mu1819	15.89	18.27	340.00	251.67
178	242	Mu1895	15.75	17.49	355.00	230.00

179	243	Mu1895	15.79	17.76	335.00	255.00
180	244	Mu1925	17.28	16.25	298.33	245.00
181	245	Mu5678	16.13	16.33	333.33	258.33
182	246	Mu11563	15.94	15.55	255.00	228.33
183	250	Mu11563	16.70	15.79	338.33	228.33
184	251	Mu11563	18.15	16.70	333.33	241.67
185	253	Mu11563	15.21	15.66	300.00	246.67
186	254	Mu11563	15.56	17.15	305.00	240.00
187	255	Mu9123	17.15	18.44	325.00	230.00
188	256	Mu9124	14.57	14.80	358.33	263.33
189	257	Mu9131	18.34	14.99	293.33	228.33
190	258	Mu9144	16.64	17.30	321.67	225.00
191	259	Mu9219	17.17	16.42	368.33	255.00
192	260	Mu9249	16.56	18.16	335.00	240.00
193	261	Mu9313	19.14	17.30	345.00	245.00
194	262	Mu9337	20.16	16.48	316.67	236.67
195	263	Mu9355	16.52	17.00	285.00	235.00
196	264	Mu9379	17.07	14.45	331.67	233.33
197	265	Mu9386	16.34	15.91	378.33	265.00
198	266	Mu9529	16.35	15.29	423.33	236.67
199	267	Mu9814	18.03	17.79	283.33	278.33
200	268	Mu9855	15.44	16.87	321.67	215.00
201	269	Mu9854	16.81	16.71	355.00	240.00
202	270	Mu9861	17.28	16.57	346.67	215.00
203	271	Mu9867	16.34	14.34	361.67	215.00
204	272	Mu9910	16.90	16.03	335.00	278.33
205	273	Mu9915	16.82	16.24	340.00	256.67
206	274	Mu9921	17.07	17.10	325.00	256.67
207	275	Mu9928	16.11	16.11	330.00	243.33
208	276	Mu9943	16.37	17.01	333.33	236.67
209	277	Mu9960	18.06	17.27	368.33	240.00
210	278	Mu3181	16.26	14.44	371.67	230.00
211	279	Mu7988	16.64	15.95	353.33	236.67
212	280	Mu7561	15.70	16.39	336.67	283.33
213	281	Mu7766	16.86	16.74	343.33	235.00
214	282	Mu3152	16.74	16.75	311.67	246.67

215	283	Mu7852	15.36	15.29	316.67	248.33
216	284	Mu9962	17.22	16.68	290.00	245.00
