# Supplementary figures and images for: Induced Genetic Variations in Stomatal Density and Size of Rice Strongly Affects Water Use Efficiency and Responses to Drought Stresses
Source: Front Plant Sci. 2022 May 25;13:801706. doi: 10.3389/fpls.2022.801706 (PMC9174926; doi:10.3389/fpls.2022.801706)

## Slide 1
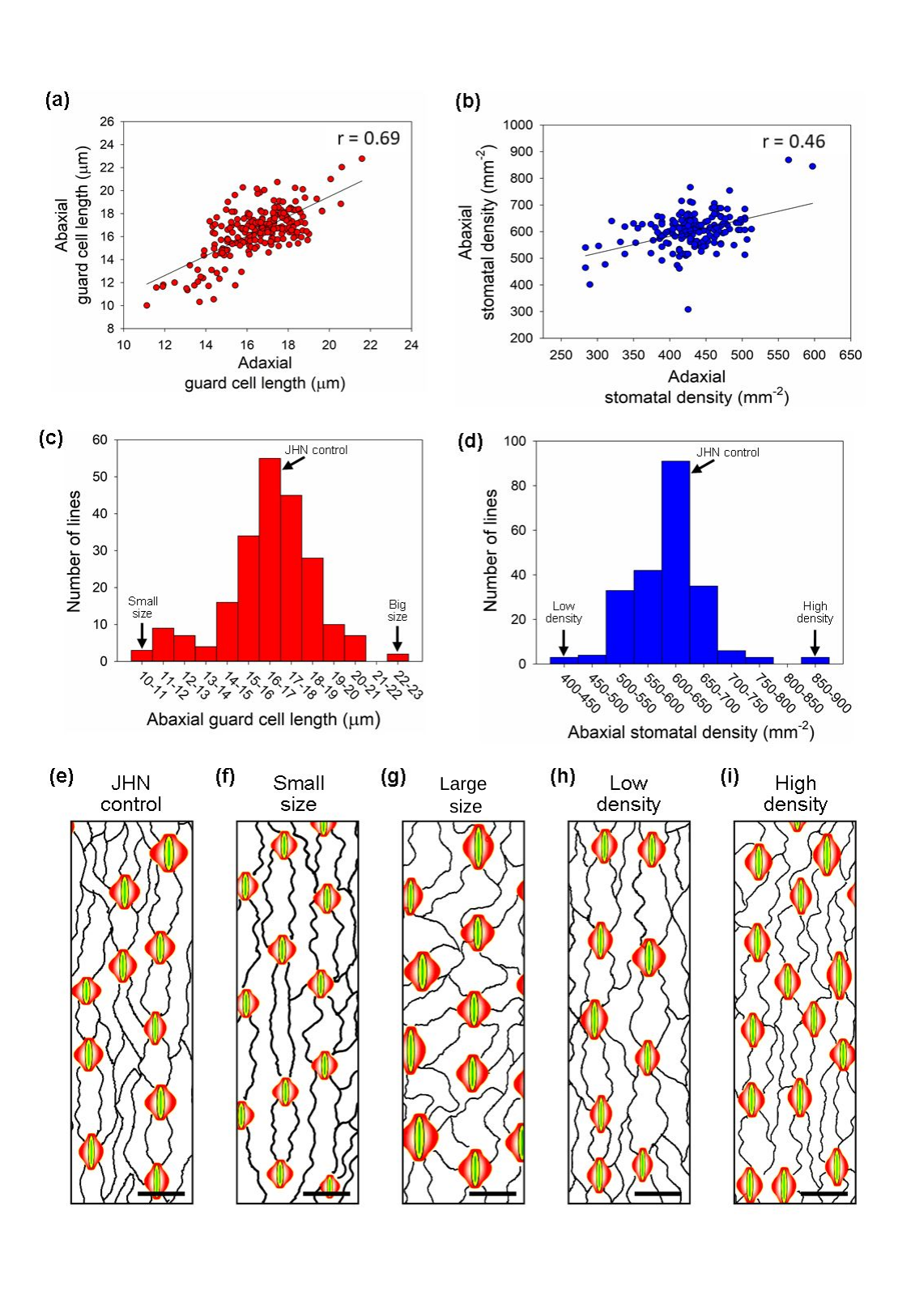

Large
size

## Slide 2
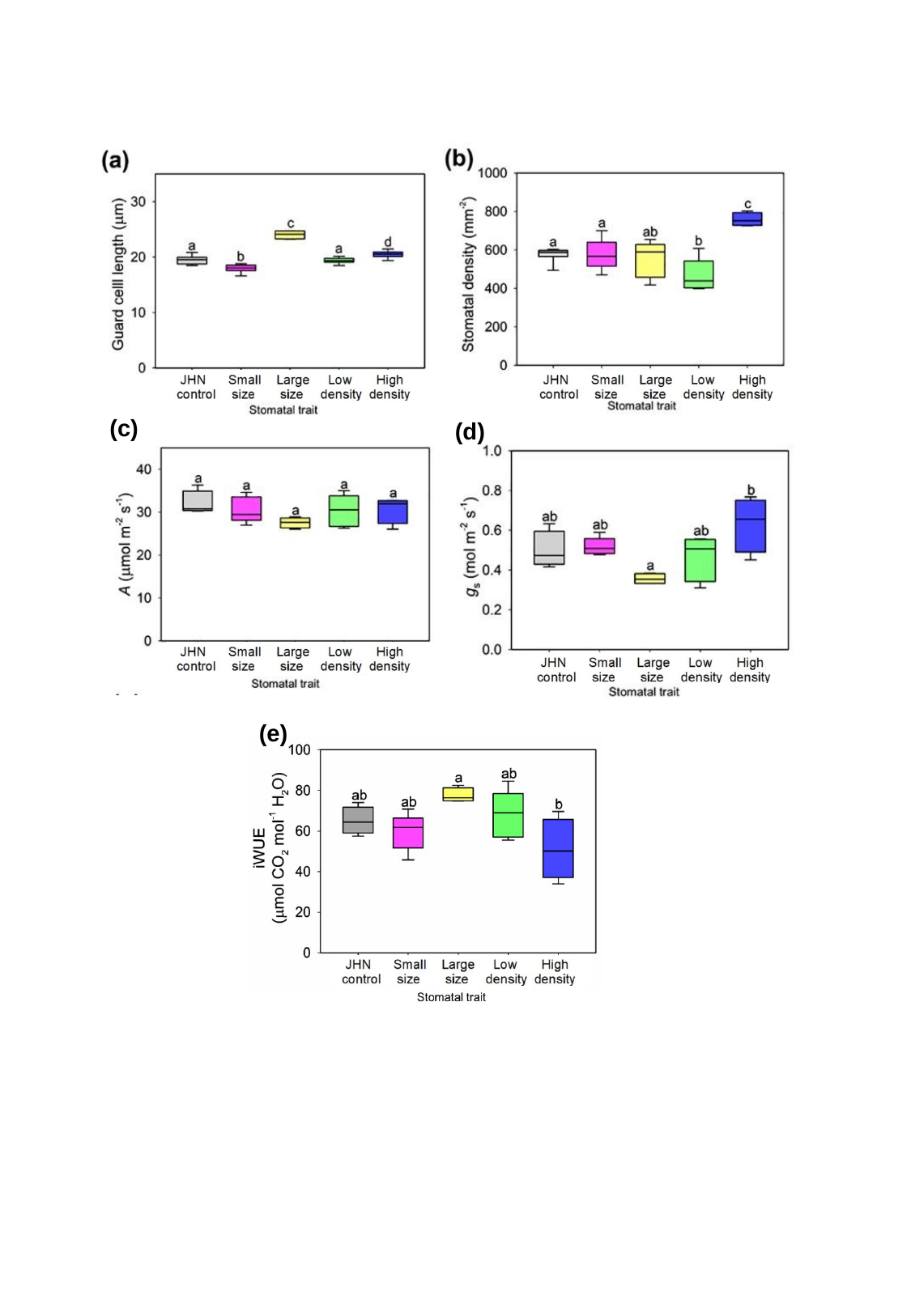

(c)
(d)
(e)

## Slide 3
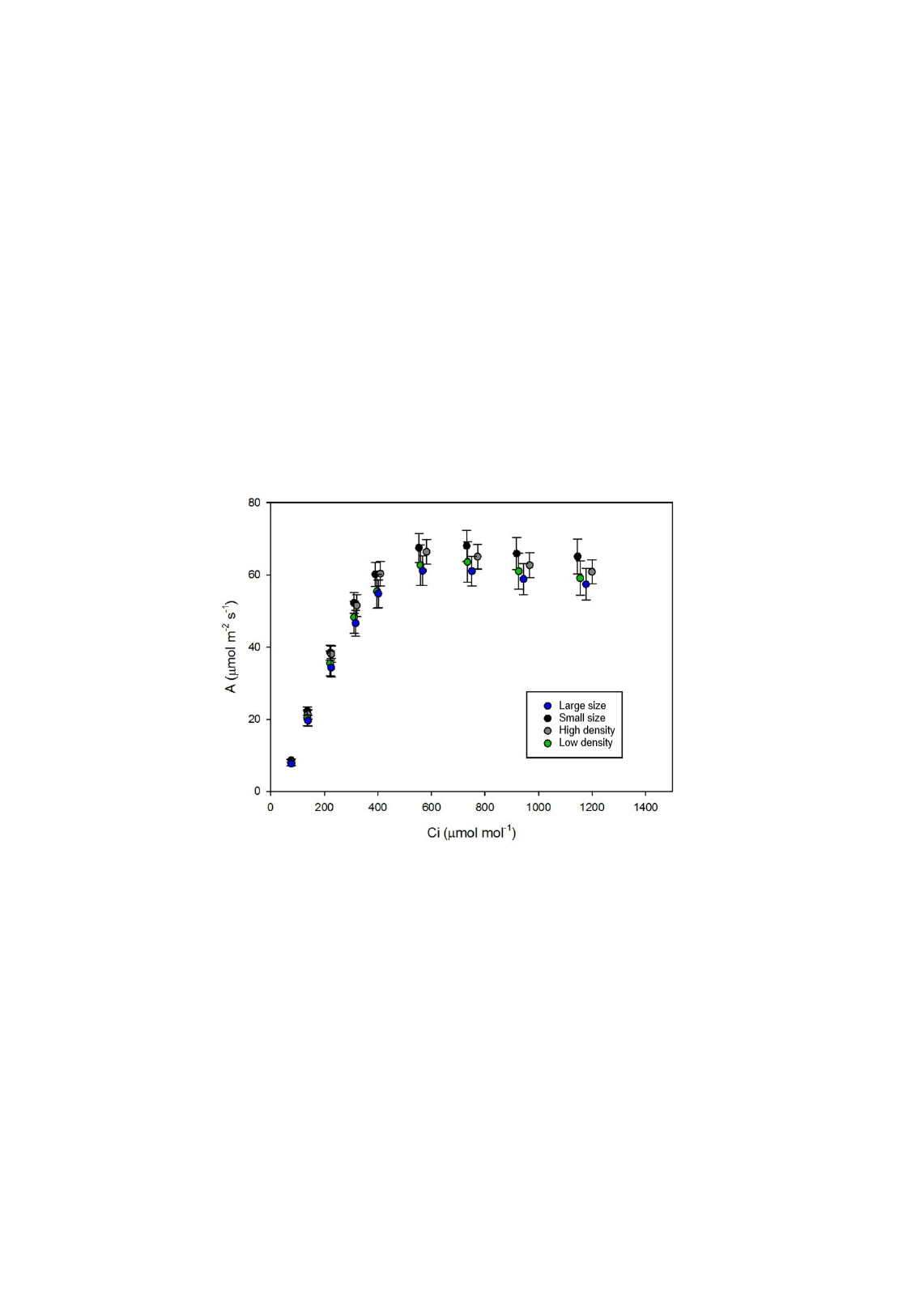

## Slide 4
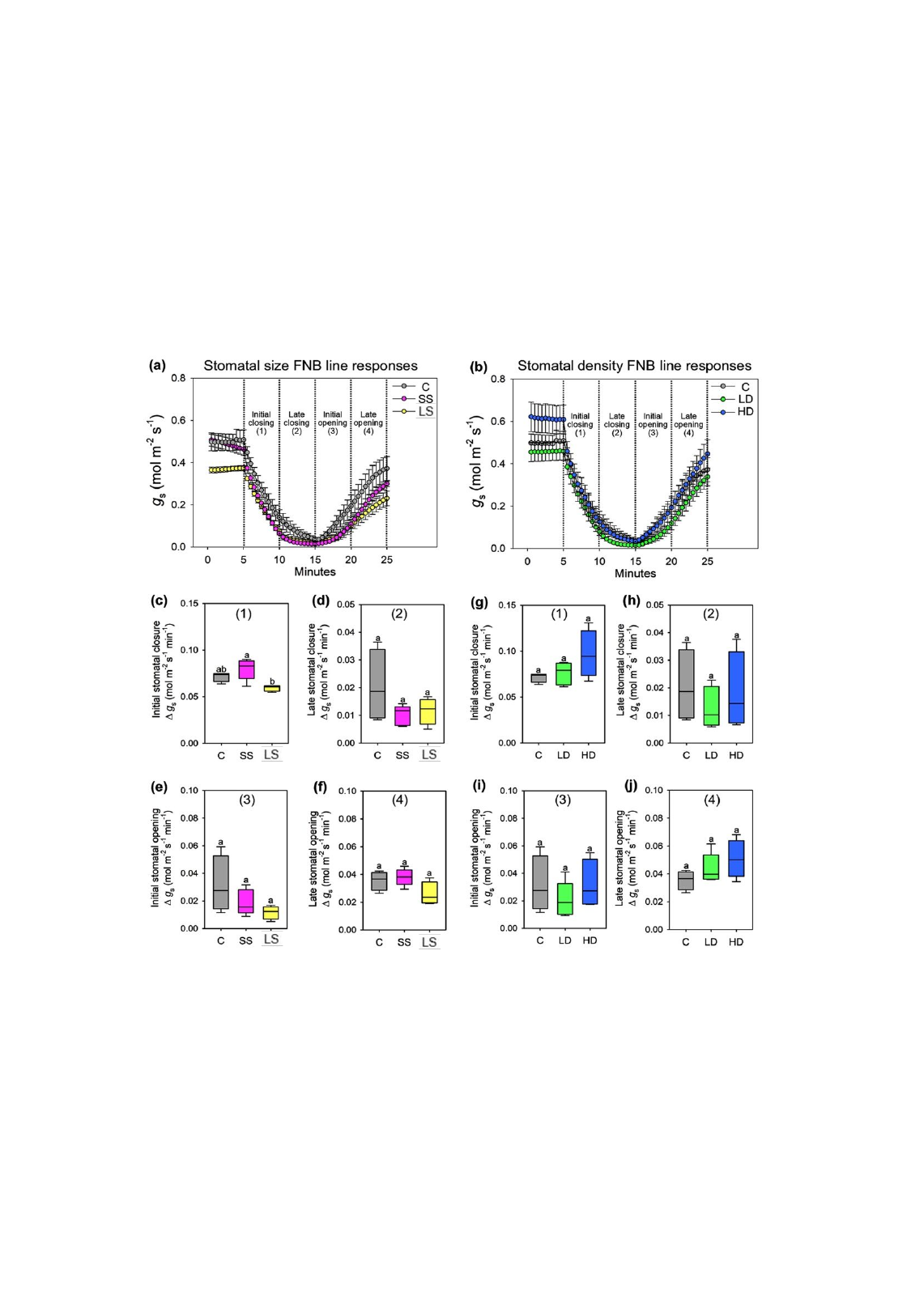

## Slide 5
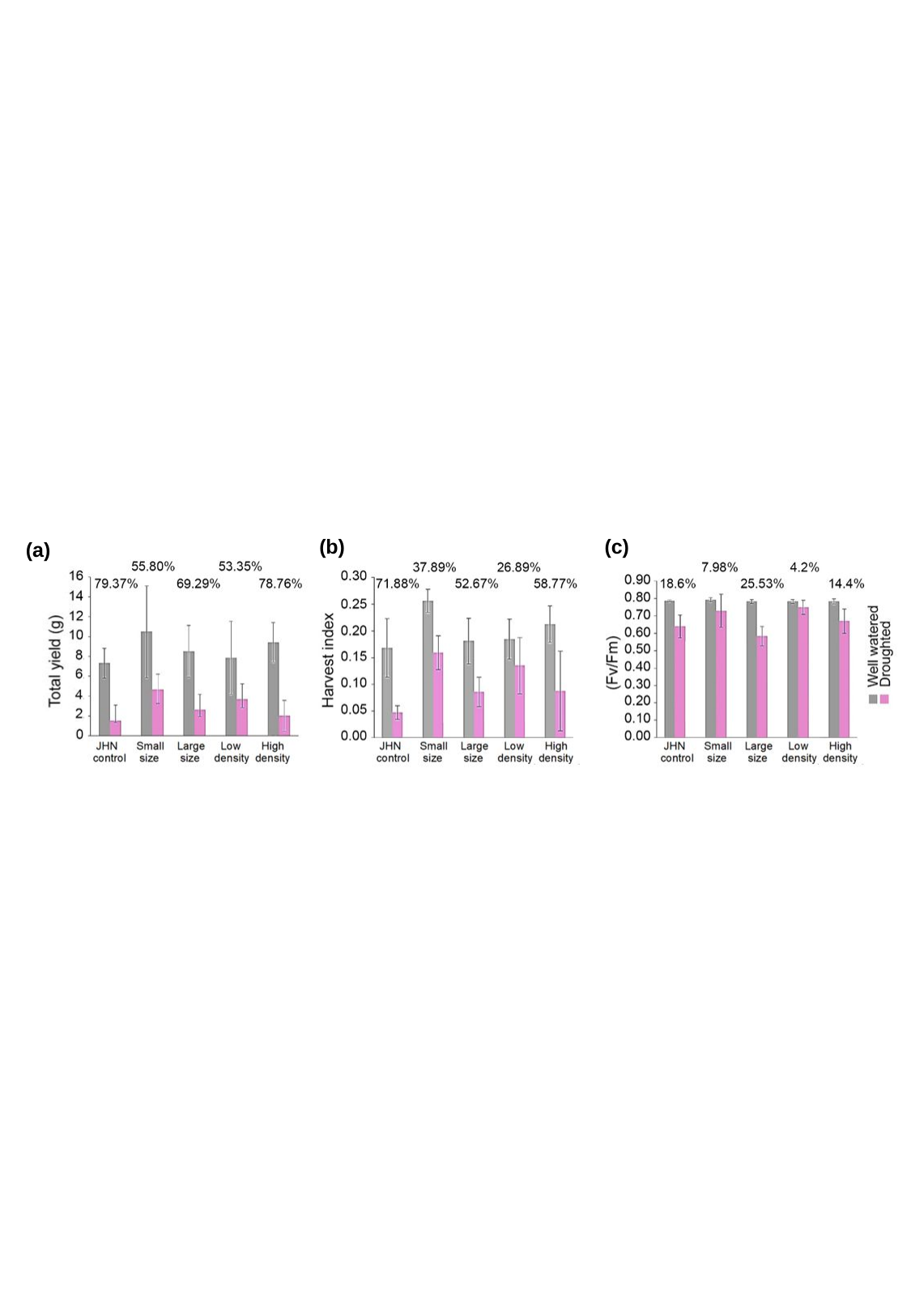

(c)
(b)
(a)

Supplement: Supplementary file 3 [file Presentation_1.PPTX]
